# Immunotherapy in triple-negative breast cancer: Insights into tumor immune landscape and therapeutic opportunities

**DOI:** 10.3389/fmolb.2022.903065

**Published:** 2022-08-19

**Authors:** Rita Ribeiro, Maria João Carvalho, João Goncalves, João Nuno Moreira

**Affiliations:** ^1^ CNC—Center for Neurosciences and Cell Biology, Center for Innovative Biomedicine and Biotechnology (CIBB), University of Coimbra, Faculty of Medicine (Polo 1), Coimbra, Portugal; ^2^ iMed.ULisboa—Research Institute for Medicines, Faculty of Pharmacy, University of Lisbon, Lisbon, Portugal; ^3^ Univ Coimbra—University of Coimbra, CIBB, Faculty of Pharmacy, Coimbra, Portugal; ^4^ CHUC—Coimbra Hospital and University Centre, Department of Gynaecology, Coimbra, Portugal; ^5^ Univ Coimbra—University Clinic of Gynaecology, Faculty of Medicine, University of Coimbra, Coimbra, Portugal; ^6^ iCBR—Institute for Clinical and Biomedical Research Area of Environment Genetics and Oncobiology (CIMAGO), Faculty of Medicine, University of Coimbra, Coimbra, Portugal; ^7^ CACC—Clinical Academic Center of Coimbra, Coimbra, Portugal

**Keywords:** triple-negative breast cancer, biomarkers, tumor mutational burden, immune gene signatures, infiltrating T lymphocytes, immunotherapy, immune checkpoint inhibitors

## Abstract

Triple-negative breast cancer (TNBC) is a clinically aggressive subtype of breast cancer that represents 15–20% of breast tumors and is more prevalent in young pre-menopausal women. It is the subtype of breast cancers with the highest metastatic potential and recurrence at the first 5 years after diagnosis. In addition, mortality increases when a complete pathological response is not achieved. As TNBC cells lack estrogen, progesterone, and HER2 receptors, patients do not respond well to hormone and anti-HER2 therapies, and conventional chemotherapy remains the standard treatment. Despite efforts to develop targeted therapies, this disease continues to have a high unmet medical need, and there is an urgent demand for customized diagnosis and therapeutics. As immunotherapy is changing the paradigm of anticancer treatment, it arises as an alternative treatment for TNBC patients. TNBC is classified as an immunogenic subtype of breast cancer due to its high levels of tumor mutational burden and presence of immune cell infiltrates. This review addresses the implications of these characteristics for the diagnosis, treatment, and prognosis of the disease. Herein, the role of immune gene signatures and tumor-infiltrating lymphocytes as biomarkers in TNBC is reviewed, identifying their application in patient diagnosis and stratification, as well as predictors of efficacy. The expression of PD-L1 expression is already considered to be predictive of response to checkpoint inhibitor therapy, but the challenges regarding its value as biomarker are described. Moreover, the rationales for different formats of immunotherapy against TNBC currently under clinical research are discussed, and major clinical trials are highlighted. Immune checkpoint inhibitors have demonstrated clinical benefit, particularly in early-stage tumors and when administered in combination with chemotherapy, with several regimens approved by the regulatory authorities. The success of antibody–drug conjugates and research on other emerging approaches, such as vaccines and cell therapies, will also be addressed. These advances give hope on the development of personalized, more effective, and safe treatments, which will improve the survival and quality of life of patients with TNBC.

## Introduction

Cancer is the second leading cause of death in the world, with 9.6 million deaths in 2018 and incidence rates growing worldwide (Bray et al., 2018). Breast cancer is the most frequent malignancy in women, accounting for one of four cancer cases, and it represents approximately 15% of all cancer deaths (Bray et al., 2018). It is a particularly stressful condition due to its associated physical, emotional, social, and economic burden (Luengo-Fernandez et al., 2013), despite the major efforts that have been made in treating the disease.

Breast cancer is a set of diseases with distinct pathological features and clinical outcomes that reflect different gene signatures and molecular patterns. Recognizing this diversity, classifications of breast tumors have emerged to aid in the diagnosis, treatment, and prognosis of the disease. Since 1968, the World Health Organization has been publishing up-to-date versions of a Histological Classification of Breast Tumors, a collection of histological and molecular pathology features for breast cancer diagnosis that is provided by experts in the field. Later, as gene expression advanced, Perou and Sorlie classified breast tumors into four subtypes: luminal A and luminal B, normal-like, and human epidermal growth factor receptor 2 (HER2)-enriched (Perou et al., 2000). A surrogate classification based on histological (hormone receptor expression) and molecular (Ki67 proliferation marker index) features is used in clinics, dividing breast cancers into five subtypes: luminal A-like, luminal B-like HER2-, luminal B-like HER2+, HER2 enriched, and triple-negative (Harbeck et al., 2019). The latter constitutes the subject of this review.

## Triple-negative breast cancer

### Epidemiology

Triple-negative breast cancer (TNBC) is a subtype that represents 15–20% of all breast tumors (Diana et al., 2018). It is more common in young pre-menopausal and African-American or Hispanic women (Morris et al., 2007).

Triple-negative tumors are particularly relevant due to their aggressiveness. TNBC is more frequently diagnosed in the advanced stages, with a preference for visceral metastasis and to a lesser extent in the bone (Klimov et al., 2017). Recurrence rates are also higher in TNBC, mostly in the first 5 years of diagnosis (Dent et al., 2007; Reddy et al., 2018). Despite their sensitivity to chemotherapy, with increased pathological complete response (pCR) rates relative to other subtypes of breast cancer (22–45 vs. 7%, respectively (Fornier and Fumoleau, 2012)), TNBC patients have higher mortality (37% mortality within the first 6 months of diagnosis (VazLuis et al., 2017)), worse cause-specific and overall survival (Li et al., 2017a) when a complete response is not achieved. This contradiction between responsiveness and poor survival is now referred to as “the paradox of TNBC,” and it drives the imperative need for new and more effective therapeutics against the disease.

### Genetic and molecular features

Triple-negative breast tumors are characterized by low expression or absence of expression of progesterone (PR) and estrogen (ER) receptors and by the lack of overexpression or amplification of human epidermal growth factor receptor-2 (HER2).

The advance in transcriptomic technologies has allowed a deeper characterization of the genetic and molecular features of triple-negative tumors, with implications for diagnosis and the search for new therapies. Gene expression profiling showed that 15–20% of women with TNBC carry BRCA1 or BRCA2 gene mutations or deficiencies, which impair DNA stability and repair, promoting carcinogenesis (Turner et al., 2004; Wong-Brown et al., 2015). Microarray assays demonstrated that 20% of TNBCs are claudin-low, characterized by genomic instability and a propensity for epithelial-to-mesenchymal transition (EMT) and thus more prone to metastization. In triple-negative tumors, 80% are basal-like (Foulkes et al., 2010), featuring the expression of proliferation-related genes (e.g., Ki67), the presence of a high number of mutations of tumor suppressor genes (TP53, RB1, and BRCA1) and of the PIK3CA oncogene (The Cancer Genome Atlas Network, 2012; Shi et al., 2018), along with the expression of proliferation and EMT-related molecules, such as cytokeratines (CK5/6, CK14, and CK17), epidermal growth factor receptor (EGFR), and vimentin (Rakha and Ellis, 2009). These properties corroborate TNBC’s aggressiveness and poor clinical prognosis (Rakha et al., 2008; Sabatier et al., 2014).

Based on this genetic and molecular heterogeneity, Lehman et al. suggested a division of TNBCs into four distinct subtypes: two highly proliferative basal-like (BL1 and BL2), a mesenchymal-like (M) involved in cell motility and EMT, and a luminal androgen receptor (LAR) enriched in hormonally regulated pathways, driven by the androgen receptor (Lehmann et al., 2011; Lehmann et al., 2016).

### Diagnosis

The diagnosis of TNBC relies on the combined data from pathology and mainly immunohistochemistry. The aforementioned subtyping of triple-negative tumors by gene expression analysis is an important tool in better understanding TNBCs’ biology, but its predictive value has not yet been established in clinical routine (Lehmann et al., 2016).

Imaging techniques alone (mammography and ultrasound) are not sufficient to distinguish TNBC from other breast cancers. However, it has been demonstrated that certain morphological features, such as circumscribed margins and the absence of calcifications, are common across TNBC tumors but atypical in other subtypes (Dogan and Turnbull, 2012).

Certain pathological attributes (tumor size, lymph infiltrate status, proliferation index, and necrosis) are well documented and enable us to distinguish between TNBC and non-TNBC as well as grade tumors. TNBCs are characterized by large lymphoplasmacytic infiltrate, stromal fibrosis and tumor necrosis, vascular and nerve invasion, and a high rate of proliferation (Rapoport et al., 2014; Abdollahi and Etemadi, 2016).

Immunohistochemistry (IHC) provides the most accurate data for TNBC diagnosis, as it allows the assessment of ER, PR, and HER2 status in patient samples (Penault-Llorca and Viale, 2012). Over the years, guidelines were written to standardize IHC techniques and the respective thresholds across laboratories. Samples are now considered ER/PR-negative when they present <1% of immunoreactive cells in IHC slides (Hammond et al., 2010), and are negative for HER2 with an IHC result of 0 or 1 for membrane protein expression (defined as no staining or weak/incomplete membrane staining) (Wolff et al., 2007). For uncertain HER2 results, the American Society of Clinical Oncology (ASCO) recommends a confirmatory analysis, performed by fluorescence *in situ* hybridization, which detects false positives and false negatives (Wolff et al., 2018). Genetic counseling and BRCA mutation status testing may also be required at the time of diagnosis, as it can influence the choice of therapeutic regimen.

### Triple-negative breast cancer therapy

#### Standard therapy and its challenges

Over the years, great advances have been made in breast cancer therapy, with proven efficacy of anti-HER2 antibodies in HER2-positive tumors (trastuzumab and pertuzumab) (Costa and Czerniecki, 2020), and targeted endocrine therapy in hormone-positive cancers (ER modulators—tamoxifen, and aromatase inhibitors—anastrazole) (Tremont et al., 2017). TNBC treatment, however, remains a challenge, especially due to the absence of the therapeutic targets expressed in other breast cancers. The heterogeneous nature of TNBC is also a problem, with different subtypes of triple-negative tumors demonstrating different sensitivities to available treatments (Yin et al., 2020). Consequently, no official recommendations have been created on how to treat TNBC. Nevertheless, the National Comprehensive Cancer Network (NCCN) in the United Stated of America and the European Society for Medical Oncology (ESMO) has published guidelines to help manage the disease (Cardoso et al., 2019; NCCN, 2020; Paluch-Shimon et al., 2020; Gennari et al., 2021).

The primary treatment approach against any localized breast tumor is surgical removal, conservative or radical, according to focality and clinical conditions, followed by adjuvant radiotherapy for locoregional management and chemotherapy. Pharmacological treatment is used as a neoadjuvant to minimize tissue removal and evaluate tumor sensitivity to chemotherapy. Adjuvant therapy can eliminate residual cancer cells and prevent relapse. Neoadjuvant treatment is particularly recommended in triple-negative tumors due to their high sensitivity to chemotherapy, especially in premenopausal women (Houssami et al., 2012).

Anthracyclines and taxanes are the main chemotherapeutic regimens against TNBC. Anthracyclines, such as doxorubicin, are molecules that inhibit topoisomerase II, blocking DNA replication and transcription and, consequently, arresting the cell cycle. Taxanes (e.g., paclitaxel and docetaxel) are antimitotic agents that inhibit cell division by affecting the stabilization of microtubules. Platinum-based compounds, such as carboplatin and cisplatin, interlink DNA strands, causing them to break and leading to cell apoptosis. This is particularly beneficial in the case of tumors that carry BRCA gene mutations, with underlying impaired DNA repairing mechanisms, and prevalent among TNBC patients. Other drugs, such as cyclophosphamide (causing DNA damage), fluorouracil, and capecitabine (blocking DNA synthesis) have also been used, particularly in combination or in sequential regimens with anthracyclines and/or taxanes or when the latter are contraindicated (Cardoso et al., 2019).

In early-stage TNBC, neoadjuvant chemotherapy is the standard of care. Triple-negative patients tend to have better responses to neoadjuvant treatments than non-TNBCs (Von Minckwitz et al., 2012), and patients that achieve pCR after neoadjuvant therapy have better survival outcomes (Huober et al., 2010). Still, only about 33% of patients present a complete response to standard neoadjuvant treatments (Cortazar et al., 2014). In TNBCs, the risk of recurrence and death is increased when the residual disease remains after the first 3 years after neoadjuvant treatment (Liedtke et al., 2008). Patients at an early stage of disease who do not receive neoadjuvant therapy should undergo adjuvant treatment (Cardoso et al., 2019). Platinum agents also have demonstrated efficacy in the neoadjuvant setting of TNBC, either as single agents (70% pCR rate) or in addition to standard neoadjuvant chemotherapy (22–75% pCR) (Sikov et al., 2015; Caramelo et al., 2019).

Adjuvant therapy with anthracyclines and/or taxanes remains the first line for advanced and metastatic TNBC (Al-Mahmood et al., 2018). Capecitabine has also been considered in the management of residual disease after neoadjuvant treatment for its good outcomes in clinical trials in terms of disease-free survival (DFS—69.8%), OS (78.8%) (Masuda et al., 2017), and pCR (33.6%) (Martín et al., 2019). In this setting, platinum therapy has failed to demonstrate a clear benefit against standard first-line anthracycline regimens (Pandy et al., 2018).

Safety remains a concern in currently available TNBC therapies due to anthracycline-induced cardiotoxicity (Barrett-Lee et al., 2009) and paclitaxel-associated hypersensitivity, neutropenia, and neurotoxicity (Patt et al., 2006). Nab-paclitaxel was developed to overcome taxane toxicity and increase the extent of tumor delivery. Nab-paclitaxel consists of a colloidal suspension of albumin-bound paclitaxel nanoparticles, a formulation that allows better pharmacokinetics and safety profile than free (solvent-based) paclitaxel (Schettini et al., 2016). The former presented a pCR of 48% in the neoadjuvant setting, in contrast with the 26% enabled by the latter (Untch et al., 2016).

However, TNBCs lack the benefit resulting from the use of targeted or hormonal systemic therapies in other subtypes. In this regard, a deeper knowledge of the molecular characteristics of TNBCs paved the way for the development of novel targeted therapeutics (Won and Spruck, 2020) and patient stratification.

#### The search for targeted therapies

Inhibitors targeting poly (ADP ribose) polymerase (PARP), an enzyme involved in DNA repair pathways, impair DNA repairing mechanisms, leading to tumor cell death. Olaparib is an inhibitor of PARP-1, PARP-2, and PARP-3 enzymes, and has been approved by the European Medicines Agency (EMA) and the Food and Drug Administration (FDA) for the treatment of patients with germline BRCA1/2-mutations, who are HER2-negative and have locally advanced or metastatic breast cancer, and who have been treated with anthracyclines and taxanes (European Medicines Agency, 2014; Food and Drug Administration, 2014). The fact that both EMA and the FDA also approved olaparib monotherapy as first-line treatment for other BRCA-mutated cancers (advanced BRCA-mutated ovarian cancer) gives hope for its use for other BRCA-deficient tumors, such as TNBC, as a first-line option (Montemorano et al., 2019). More recently, talazoparib, an inhibitor of PARP-1 and PARP-2 enzymes, was approved by both FDA (2018) and EMA (2019) as an alternative for patients with germline BRCA mutations and HER2-negative locally advanced or metastatic breast cancer who have been previously treated with anthracycline and/or taxane (Food and Drug Administration, 2018; European Medicines Agency, 2019). Veliparib and niraparib are PARP-1 and PARP-2 inhibitors that are under clinical investigation for the treatment of TNBC (Geenen et al., 2018). Velaparib demonstrated efficacy when used in combination with EGFR-inhibitor lapatinib (Stringer-Reasor et al., 2021) or cisplatin (Sharma et al., 2020) but did not improve pCR when added to a standard neoadjuvant plus carboplatin regimen (Loibl et al., 2018). Niraparib’s clinical activity was evaluated in a phase II trial in combination with pembrolizumab immune checkpoint inhibitor (Vinayak et al., 2019) and a phase III trial as monotherapy against standard chemotherapy (Turner et al., 2021). However, due to the small sample sizes in the trials and information-censoring issues, no accurate conclusions could be drawn about its effectiveness in TNBC treatment.

Because a large percentage of triple-negative tumors have been identified as basal-like and thus have a high proliferation index, antimitotic agents (taxanes) are considered a targeted therapy. In this respect, response markers are important for determining which patients will benefit the most from taxane therapy. A microtubule-associated protein (Bauer et al., 2010) and mitotic and ceramide metagenes (Juul et al., 2010) are examples of markers that are associated with higher pCR levels in patients treated with neoadjuvant paclitaxel with basal-like-TNBC than in other triple-negative subtypes or non-triple-negative tumors. Further, as previously mentioned, many basal-like tumors express EGFR and thus are a potential target for EGFR inhibitors such as cetuximab. This has been tried against basal-like TNBC in clinical trials, either alone or in combination with chemotherapy (paclitaxel or carboplatin), although with only modest efficacy (Costa et al., 2017).

Angiogenesis inhibitors are another approach against TNBC, as the overexpression of VEGF in these tumors is higher than it is in non-TNBC (Linderholm et al., 2009). Bevacizumab, an anti-VEGF antibody, has shown moderate results in a neoadjuvant setting, either as first-line (Miles et al., 2013) or in combination with chemotherapy (Bell et al., 2017). In advanced and metastatic tumors, so far, it has failed to demonstrate a robust benefit (Manso et al., 2015). In 2008, FDA approved the use of bevacizumab in combination with paclitaxel for the treatment of metastatic breast cancer. However, in 2010, bevacizumab’s indication for breast carcinoma was withdrawn, after it was shown not to be safe and effective in this indication (Food and Drug Administration, 2019). In Europe, the use of bevacizumab is still in place, combined with paclitaxel or capecitabine for the first-line treatment of metastatic breast cancer.

Due to the highly proliferative nature of triple-negative tumors, inhibitors of the PI3K/Akt/mTOR pathway, which regulates the cell cycle, are also considered as potential anti-TNBC therapies (Khan et al., 2019). This is based on demonstrations that mutations in PI3K are more prevalent in TNBC than in other breast cancers (Koboldt et al., 2012), as well as on the activation of the mTOR pathway and its correlation with poor prognosis (Pelicano et al., 2014). Ipatasertib is a PI3K inhibitor that showed modest but positive efficacy as neoadjuvant therapy in PTEN-mutant patients when used in combination with paclitaxel by demonstrating a progression-free survival (PFS) of 6.2 vs. 3.7 months in the group receiving paclitaxel as monotherapy (Kim et al., 2017), and a pCR of 16 vs. 13%, respectively (Oliveira et al., 2019). Everolimus is an m-TOR inhibitor that has been tested in combination with carboplatin (Singh et al., 2014), liposomal doxorubicin/bevacizumab (Basho et al., 2018), gemcitabine/cisplatin (Park et al., 2018), and cisplatin/paclitaxel (Jovanovic et al., 2017). Despite moderate efficacy, the concerns that emerged regarding hematological toxicity (neutropenia and thrombocytopenia) demand further research.

Patients with the LAR subtype of TNBC, enriched in androgen receptor expression and hormonally regulated pathways, may benefit from anti-androgen therapy. Androgen receptor inhibitors (enzalutamide and bicalutamide) (Gucalp et al., 2013; Traina et al., 2018), androgen synthesis inhibitors (abiraterone acetate) (ClinicalTrials.gov, 1842), and a combination of androgen inhibitors with PI3K inhibitors (Lehmann et al., 2014) are among the approaches currently receiving clinical attention.

#### Chemoresistance: A real threat

Chemoresistance is a growing concern in TNBC therapy, with about 30–50% of patients undergoing neoadjuvant therapy evolving to resistant recurrences, resulting in poor outcomes (Kim et al., 2018). Mechanisms of resistance arise when tumor cells are exposed to cytotoxic agents, as a means of maintaining their viability. Some of these mechanisms have been demonstrated for TNBC standard therapies, and strategies to overcome them have been proposed (O’Reilly et al., 2015).

It was previously demonstrated that resistance to anthracyclines is associated with reduced expression or function of the target DNA repair enzyme, topoisomerase II (Nielsen et al., 1996), while taxane resistance has been linked to β-tubulin III overexpression (Tommasi et al., 2007).

ATP-binding cassette (ABC) transporters are a family of transmembrane proteins that promote drug efflux, preventing its action in the cell. In TNBC cells, the three ABC transporters responsible for resistance to anthracyclines and taxanes have been identified: multidrug-resistant protein-1 (MRP1), breast cancer resistance protein (ABCG2), and P-glycoprotein (MDR1) pump (Scharenberg et al., 2002; Leonessa and Clarke, 2003). Attempts to hinder ABC resistance have been studied for several cancers, including breast cancers. The strategies used include inhibition of ABC activity (with non-steroidal anti-inflammatory drugs (O’Connor et al., 2007) or tyrosine kinase inhibitors (Nakai et al., 2016)) and expression (with microRNA (Wang et al., 2015)).

##### The stemness phenotype

A strong hypothesis that could explain the emergence of chemoresistant breast tumors is the maintenance of a minor quiescent population, namely, cancer stem cells (CSC). It has been demonstrated that TNBC patient tissues and cell lines present a higher abundance of CD44+/CD24-stem cells, as well as EMT-related gene signatures, than other subtypes, in line with the stemness phenotype (Park et al., 2010; Zhou et al., 2016; Shibue and Weinberg, 2017). Evidence of this phenomenon also comes from an increase in RNA transcripts, the expression of TGF-β1 and TGF-β type 1 receptors, and molecules associated with CSCs and EMT mechanisms in TNBC tumor biopsies after chemotherapy (Bhola et al., 2013). Strategies to circumvent this resistance may include selective inhibitors of CSC (Gupta et al., 2009), antagonists of CSC membrane markers (e.g., CD44), or targeting of TGF-β signaling (O’Conor et al., 2018).

Hypoxia-inducible factors (HIFs) are transcription factors that maintain oxygen homeostasis in the cell. HIF expression is increased in tumors, in response to the insufficient supply of oxygen resulting from the rapid and disordered neo-angiogenesis (Hanahan and Weinberg, 2011). In TNBC, HIFs have been associated with the CSC phenotype, chemoresistance, and poor prognosis (Jin et al., 2016; Xiong et al., 2018). In fact, the co-administration of HIF inhibitors and chemotherapy have been demonstrated to overcome CSC-mediated resistance, as exemplified by the ability of digoxin to reverse resistance to paclitaxel and gemcitabine in mouse models of TNBC (Samanta et al., 2014). In spite of the search for molecular targets involved in hypoxia to overcome chemoresistance, no clinical benefit has been reported so far in this context (Soni and Padwad, 2017).

##### Genomic diversity

The genomic diversity characteristic of TNBC could also explain the appearance of chemotherapy-resistant relapses. In 2018, Santonja et al. correlated the different subtypes of TNBC with patient responsiveness and resistance to treatment. The LAR subtype had lower pCR rates, suggesting higher resistance to standard chemotherapy, in line with its predicted responsiveness to endocrine therapy (Santonja et al., 2018). This genomic diversity is also reflected in genes that are often mutated in TNBC patients and correlated with apoptosis avoidance, a drug-resistance mechanism (e.g., the p53 (Lin et al., 2019) and Bcl-2 genes) (Inao et al., 2018). The higher frequency of mutations found in treated TNBCs relative to naive tumors may support the hypothesis that certain gene alterations, such as in tumor suppressor genes (e.g., p53, and PTEN), or those associated with cell proliferation, such as MYC, likely form the basis for the development of chemoresistance (Koboldt et al., 2012; Balko et al., 2014).

Finally, the signaling pathways involved in cell growth and proliferation may be implicated in TNBC chemoresistance. NF-κB regulates the transcription of genes involved in TNBC progression (Poma et al., 2017) and chemoresistance in breast cancers (Fan et al., 2008). As mentioned, the PTEN/PI3K/AKT/mTOR pathway is often hyperactivated in TNBC and has been associated with chemoresistance in breast cancer (Steelman et al., 2008). The JAK/STAT pathway consists of a phosphorylation cascade of proteins that regulates the transcription of genes involved in tumorigenesis, survival, and anti-apoptosis. Because TNBCs enriched in JAK amplifications have been associated with the worst prognosis, it is hypothesized that this pathway may be also involved in chemoresistance (Balko et al., 2014). Different STAT proteins have been associated with distinct outcomes in TNBC, with STAT3 and STAT5 activation being linked to chemoresistance and their inhibition to therapy sensitivity (Furth, 2014; Mumin et al., 2019).

The lack of therapeutic targets, along with the rise of chemoresistance, reinforces the need for the development of new approaches against TNBC.

## Triple-negative breast cancer: An immunogenic subtype

The microenvironment of breast tumors is composed of not only cancer cells but also of a variety of other cellular and non-cellular components, such as endothelial cells, immune cells, fibroblasts and adipocytes, the extracellular matrix, and chemical mediators. The composition of the tumor microenvironment differs among the various breast cancer subtypes and understanding it is important to improve diagnosis and provide more effective treatment of TNBC (Yu and Di, 2017).

The immune component of tumors has already been intensively studied, particularly since 2011, when Hanahan and Weinberg suggested that it is involved in carcinogenesis, proposing the ability of tumors to evade attack and elimination by the immune system as a revised hallmark of cancer (Hanahan and Weinberg, 2011). The work of Dunn et al. contributed to the understanding the crosstalk between tumors and the immune system is much more complex, for both immunodeficient and immunocompetent individuals, by recognizing that, not only do tumors have mechanisms for escaping immunologic defenses but they can also be shaped by their immune surroundings, in a process named immunoediting (Dunn et al., 2004). These findings are particularly important for immunotherapies because it has been observed, although the reason has not been clearly understood, that only a small subset of patients benefit from these therapeutics. For example, in 2018, only about 13% of patients enrolled in checkpoint inhibitors treatments in the United States were responsive to treatment (Haslam and Prasad, 2019).

In the tumor microenvironment, both innate and adaptive immune responses are triggered. Innate immune cells include antigen-presenting cells (APC), macrophages, neutrophils, and monocytes. These constitute the first response to foreign elements and help the adaptive immune cells, i.e., T and B lymphocytes, recognize the neoantigens expressed in tumor cells. T lymphocytes are a set of cells with distinct roles that can be differentiated by their cell surface markers. CD8^+^ lymphocytes are cytotoxic T cells that recognize tumor antigens and eliminate malignant cells by releasing pro-inflammatory cytokines, such as interferons (INF) and interleukins (IL), as well as granzyme–perforin complexes. CD4^+^ T lymphocytes are helper cells that can differentiate into Th1 or Th2 cells. Th1 cells secrete INFα, INFγ, and IL-2, which are able to activate macrophages and NK cells against tumor cells and are thus predictive of a good prognosis. On the other hand, Th2 cells produce and release anti-inflammatories IL-4, IL-5, and IL-10, which promote tumor growth and metastasis. Regulatory T cells (Tregs) are a class of lymphocytes that express forkhead box P3 (FOXP3) transcription factor, and CD25 surface marker. They have immunosuppressive activity, thus being correlated with a worse prognosis (Gajewski et al., 2013; Lee et al., 2019). T lymphocytes, like tumor cells, express immune inhibitory programmed cell death receptor-1 (PD-1), its ligand programmed death ligand-1 (PD-L1), and cytotoxic T-lymphocyte-associated protein 4 (CTLA-4), which is responsible for suppressing immune activity (He and Xu, 2020). Consequently, the immune system is either a promoter or suppressor of tumor growth, depending on the equilibrium of existing immune cells and cytokines. Its understanding and modulation have changed the cancer treatment paradigm.

In this regard, the gene signatures of immune cells (gene-transcription and proliferation related genes) and tumor-infiltrating lymphocytes (TILs) have major implications for tumor development, clinical response, and prognostic value. This knowledge may be particularly important for tumors that have limited treatment options and worse prognoses, such as TNBC. Although breast cancers are not as immunogenic as other solid tumors that have benefited from immunotherapies, such as renal cancer, non-small cell lung cancer (NSCLC), or melanoma, evidence shows that TNBC and HER2+ are more immunogenic than the hormone-positive subtypes, and the study of the TNBC immunologic landscape has provided valuable information on immunogenicity and immune activity (Liu et al., 2018).

The advancement of sequencing technologies, such as whole genome sequencing (WGS), next generation sequencing (NGS), and RNA-seq profiling has been an important contribution to the growing and clearer perception of the role of tumor immune microenvironment in cancer development, treatment, and prognosis.

## The importance of immune biomarkers

### Tumor mutational burden

**FIGURE 1:**
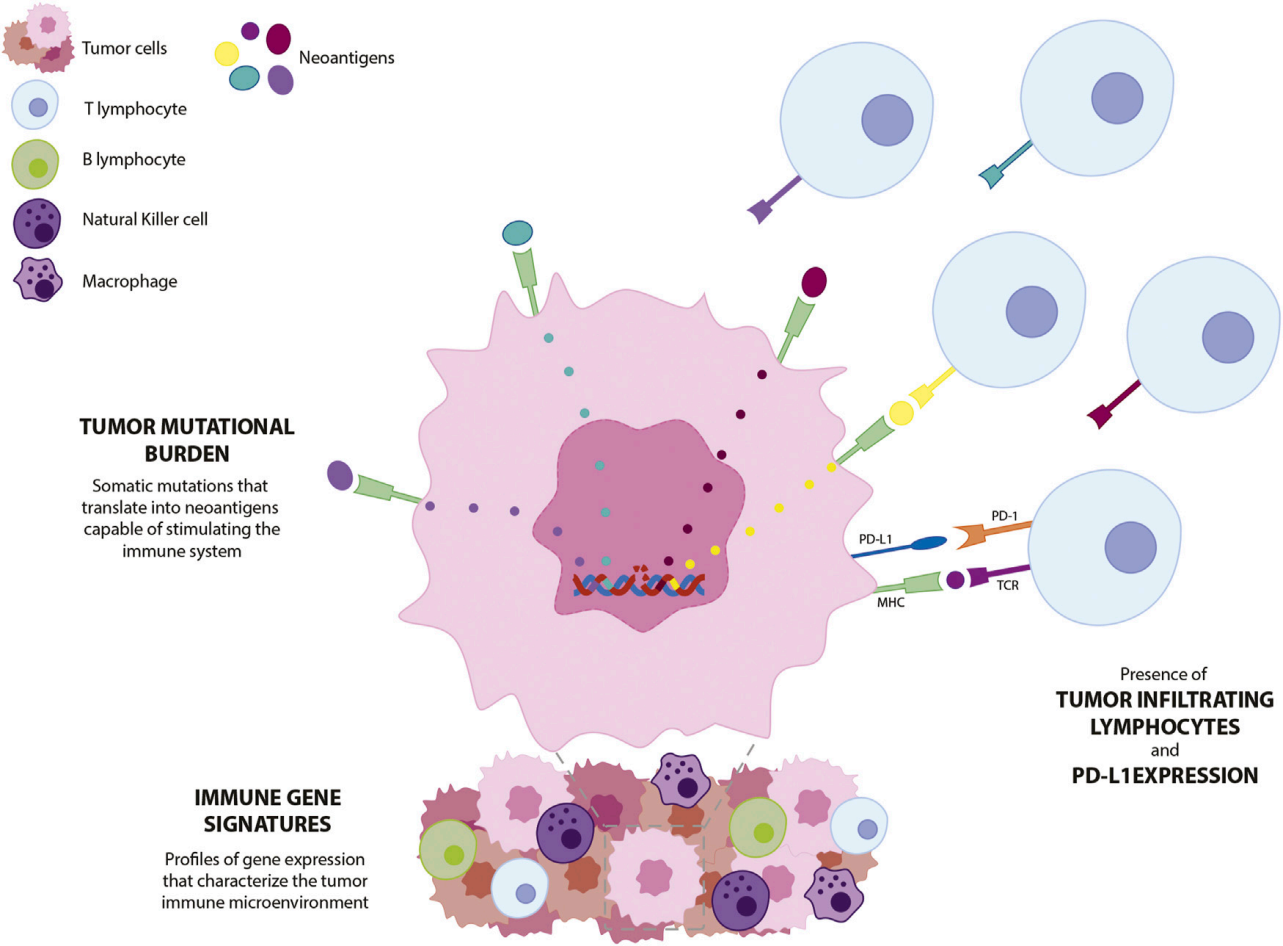
Schematic representation of biomarkers currently explored in TNBC. Point mutations in tumor cells translate into expression of tumor-specific neoantigens. Neoantigens induce T lymphocyte infiltration and increased expression of PD-1 and PD-L1 checkpoint molecules. Gene profiling of tumor biopsies allows the characterization of the immune components of the tumor. PD-L1—programmed death-ligand 1; PD-1—programmed cell death protein 1; MHC—major histocompatibility complex: TCR—T-cell receptor.

Tumor immunogenicity refers to the ability of a tumor to elicit an immune response. This depends mostly on the relative and absolute density of the antigens capable of activating the immune system, whether they are shared antigens (already present in normal tissues but overexpressed in tumor cells) or tumor-specific neoantigens (mutated proteins in tumors, not present in normal tissues) (Blankenstein et al., 2012). Neoantigens are a consequence of non-synonymous somatic mutations that result in peptides or proteins expressed at the surface of tumor cells but not of normal cells, which makes them an ideal target for immunotherapy, as represented in Figure 1.

Determination of the number of non-synonymous somatic mutations occurring in a tumor (mutations/megabase), i.e., the tumor mutational burden (TMB), demonstrated that neoantigens generated by point mutations in normal genes may be tumor specific. This is very important for breaking tumor heterogeneity and for clustering patients with similar genetic signatures, thus enabling superior patient stratification to benefit from a certain treatment for use as a predictive of therapy response (Samstein et al., 2019). In fact, TMB is predictive of the response to immunotherapy in a variety of cancers, as different tumors with higher TMB values showed an increased objective response rate (ORR) when treated with immunotherapies (Yarchoan et al., 2017; Loibl et al., 2019). Additionally, the establishment of TMB as a predictive factor in immunotherapy has been recently validated by FDA’s approval of checkpoint inhibitor pembrolizumab for patients with solid tumors presenting high TMB (defined to have ≥10 mutations/megabase) (FDA, 2020c). This agnostic approval was based on the results of the KEYNOTE-158 study that concluded that high TMB is predictive of pembrolizumab’s efficacy against a variety of advanced solid tumors, such as small cell lung cancer, mesothelioma, and neuroendocrine, biliary, vulvar, and anal cancers (Marabelle et al., 2020).

Although breast cancer is not among the group of cancers with higher TMBs, hormone-negative tumors such as TNBC and HER2+ were observed to have significantly higher TMB than other subtypes (Barroso-Sousa et al., 2020). In this regard, an association between TMB and immune-mediated patient survival was demonstrated for patients with a favorable immune-infiltrate disposition, while patients with high TMB and poor immune-infiltrates showed a worse prognosis (Thomas et al., 2018). In another study, a better prognosis was also correlated with lower TMB and neoantigen counts, possibly due to the lower clonal heterogeneity due to immunosurveillance mechanisms, which eliminate cells with higher neoantigen expression (Karn et al., 2017). Therefore, the potential value of TMB as a biomarker for triple-negative breast tumors is still controversial, and its clinical role is far from being significant.

To improve the value of TMB as a biomarker in cancer therapy, further investigation is needed, including studies that have larger sample sizes, standardized sample treatments and sequencing parameters, and harmonization of clinical output reporting. The organizations Friends of Cancer Research and the Quality Assurance Initiative Pathology published a set of recommendations to improve TMB estimation and report in the clinics (Stenzinger et al., 2019).

### Immune gene signatures

Immune gene signatures are the profiles of gene expression used to characterize the immune response in tumors. Transcriptomic techniques and in-depth statistical analysis are used in cancer cell lines or patient-derived tumor samples to identify clusters of genes whose expression is involved in the immune response to cancer, as well as to characterize the immune cell composition of tumors (Figure 1).

For breast cancer, the regulatory authorities have approved four commercial multi-gene expression assays with prognostic value, Prosigna^®^ (PAM50), MammaPrint^®^, Oncotype DX^®^, and Endopredict^®^ (Cardoso et al., 2019), which are based on gene signatures that are reflected in the tumor phenotype and its response to its environment. Specifically regarding the immune response in breast cancer, no clinical signatures have yet been made available. Nevertheless, the genes involved in the immune response to cancer and the underlying prognostic value have been identified. Finak *et al.* demonstrated that two gene clusters from a set of 163 genes were correlated with good or bad prognosis in breast cancer, termed the stroma-derived prognostic predictor (SDPP; Table 1). Good outcomes were related to the enrichment of gene coding for T lymphocyte and NK cell activation, as well as to granzyme activity, suggesting an immune response to the tumor. Genes associated with bad outcomes (higher risk of recurrence or death from the disease) code for molecules involved in carcinogenic mechanisms, such as angiogenesis, hypoxia, EMT, and tumor-associated macrophages (immunosuppression and metastasis) (Finak et al., 2008).

**TABLE 1:** Immune gene signatures that characterize antitumor immune response, including pan-cancer signature, breast cancer signatures, and TNBC signature. Gene signatures include genes involved in immune cell activation and proliferation, and expression of molecules that regulate the immune response, such as chemokines and interferons.

Immune gene signature	Gene	Immune function	Reference
Pan-cancer immune gene signature	*CD84*	Cytotoxic T cells	Liu et al. (Liu, 2019)
	*CD79A*	B cells	
	*EVI2B*	Neutrophils	
	*GATA3* *STAT*	T-helper cell regulation	
	*FOXP3*	T regulatory cells	
	*CD68*	Macrophages	
	*KLRC1*	NK cells	
	*LILRA4*	Dendritic cells	
	*TBX21*	IFNγ production	
SDPP signature (good prognosis)	*CD8A*, *CD247*, *CD3D*	T cells	Finak et al. (2008)
	*CD8A*	MHC class I protein binding	
	*GZMA*, *GZMB*	Granzymes A and B	
SDPP (bad prognosis)	*GIMAP5*	T cells	
	*ADM*	Adrenomedullin	
	*CXCL14*	NK cells activity	
	*IL8*	Interleukin-8	
	*EDN1*	Endothelin-1	
	*SPP1*	Osteopontin	
	*CXCL1*	Chemokine ligand	
Immune response module	*STAT1*, *STAT4*	Lymphocytes activity	Desmedt et al. (2008)
	*CD69*	T cells	
	*CD48*	B cells	
	*CXCL9*, *CXCL10*, *CXCL11*, *CCL5*, *CCL8*	Chemokine ligands	
Immune response and regulation signature	*CD27*, *CD52*	T cells	Yang et al. (2018)
	*GZMA*, *GZMK*	Granzymes	
	*CCR2*	Chemokine receptor	
	*CCL5*, *CXCL9*	Chemokine ligands	
Four genes immune signature in TNBC	*CXCL13*	Chemokine ligand	Criscitiello et al. (2018)
	*GBP1*	Interferon-induced protein	
	*SULT1E1*	Immunogenic protein	
	*HLF*	Immunogenic cell death	

CD, Cluster of Differentiation; EVI, Ecotropic Viral Integration Site; GATA, GATA binding protein; STAT, Signal Transducer and Activator of Transcription; FOXP3, Forkhead box P3; KLRC1, Killer Cell Lectin Like Receptor C1; LILRA4, Leukocyte Immunoglobulin Like Receptor A4; TBX21, T-Box Transcription Factor 21; GZM, Granzyme; GIMAP5, GTPase, IMAP Family Member 5; ADM, Adrenomedullin; CXCL, Chemokine Ligand (C-X-C motif); CCL, Chemokine Ligand (C-C motif); IL, Interleukin; EDN, Endothelin; SPP1, Secreted Phosphoprotein 1; CCR, Chemokine Receptor; GBP1, Guanylate Binding Protein 1; SULT1E1, Sulfotransferase Family 1E Member 1; HLF, Hepatic leukemia factor.

Some signatures are tumor-specific, which can help differentiate immune mechanisms across cancer subtypes and potentially lead to patient stratification and targeted treatment. For TNBCs, these immune signatures may have particular importance due to the absence of common molecular features in breast cancer (ER, PR, and HER2 expression). Desmedt *et al.* studied immune gene signatures for more than 2000 breast cancers and associated the different subtypes with different biological processes (tumor invasion/metastasis, immune response, angiogenesis, evasion of apoptosis, growth and proliferation, and ER and HER2 signaling), and each of these with a prognosis value (Table 1). In this study, only ER/HER2 tumors presented a clinical outcome that was significantly associated with the immune response module, where the high expression levels of these genes were correlated with increased relapse-free survival (Desmedt et al., 2008). Yang *et al.* used a 17-gene immune signature to identify an immune-enhanced group of breast cancers, with characteristics of ER- and claudin-low tumors, which was correlated with better clinical outcomes (lower risk of recurrence, metastasis, or death) (Yang et al., 2018) (Table 1).

As patients responding to neoadjuvant therapy tend to have longer disease-free survival, and chemotherapy is hypothesized to activate the tumor's immune system in breast cancer (Kroemer et al., 2015), immune gene signatures that are predictive of prognosis after neoadjuvant treatment may become particularly relevant to clinical practice. Regarding the immune response after therapy, studies in samples from patients after neoadjuvant therapy correlated immune signatures with recurrence-free survival (Rody et al., 2011), increased pCR (Ignatiadis et al., 2012; Lee et al., 2015), and prolonged survival (Stoll et al., 2015; Hendrickx et al., 2017). Criscitiello *et al.* assessed gene immune signatures specifically in TNBC patients who were previously treated with neoadjuvant therapy. *CXCL13* (chemokine), *GBP1* (interferon response), *SULT1E1* (estrogen homeostasis, potentially immunogenic), and *HLF* (immunogenic cell death) were correlated with chemotherapy-induced immune response and increased distant relapse-free survival (Criscitiello et al., 2018) (Table 1).

Importantly, many of these immune signatures show little overlap, which hinders their usefulness for prognosis or therapeutic choice in clinical practice. Further research is needed, including large clinical trials designed to identify and validate gene markers, to facilitate the translation from the laboratory to the clinic.

### Tumor infiltrating lymphocytes and PD-L1 expression

Notwithstanding that healthy breast tissue has a low number of immune cells, tumor development is associated with leucocytes infiltrating the area, with B and T lymphocytes, Tregs, and neutrophils representing the main immune populations (Althobiti et al., 2018).

Tumors with highly proliferative characteristics, such as the triple-negative and HER2-positive subtypes, present increased levels of TILs. In TNBC, in particular, this is explained by its increased genomic instability and mutational burden, with consequent stimulation of the immune system for elimination of cells bearing non-self-antigens (Smid et al., 2011) (Figure 1). For these breast cancer subtypes, the presence of immune infiltrates is associated with a good prognosis in patients treated with standard neoadjuvant chemotherapy or trastuzumab (for HER2-overexpressing tumors) (Denkert et al., 2018; Ochi et al., 2019).

Triple-negative tumors are also enriched in both CD8^+^ T cells and T reg cells relative to other breast cancer subtypes (Stanton et al., 2016). As expected, the presence of cytotoxic T cells is associated with a good prognosis in TNBC in the early stage of the disease (Blackley and Loi, 2019), and with response prediction to neoadjuvant (Denkert et al., 2018) and adjuvant chemotherapy (Adams et al., 2014; Pruneri et al., 2016). In addition, the low area fraction of stromal TILs and deficiency of CD8^+^ cells are indicative of an increased risk of mortality (Vihervuori et al., 2019). Unexpectedly, T regulatory cells were found to be predictive of good prognosis in TNBC, despite their role in suppressing the activity of immune cells. TNBC patients who had higher expression of FOXP3 had higher OS and PFS than TNBC patients with lower levels of FOXP3 (Lee et al., 2012; Jiang et al., 2015; Yeong et al., 2017). However, these results may be misleading, as there is a need for optimization of T reg cells identification. This was exemplified by a study correlating FOXP3/CD25+ Treg cells with improved OS in TNBC patients but not Tregs identified by the FOXP3 marker alone (Zhang et al., 2019).

An important source of immune cells in the tumor microenvironment is the presence of tertiary lymphoid structures (TLS). These vascularized clusters of lymphoid cells have a similar structure and function to secondary lymphoid organs, as they are mainly composed of T cells, dendritic cells, plasma cells, and B cells, promoting cellular and humoral anti-tumor response (Wang et al., 2022). Therefore, TLS constitute an opportunity for immunotherapeutic approaches, not as predictors of outcome, but as potentiators of immunotherapies (Sautès-Fridman et al., 2019). In TNBC, the presence of TLS was correlated with higher DFS and OS (Lee et al., 2016), and increased pCR in patients treated with neo-adjuvant chemotherapy (Song et al., 2017).

It is important to emphasize that metastasis from TNBC primary tumors is less immunogenic than the latter, as they present inferior levels of infiltration of CD8^+^ lymphocytes and PD-1-positive T lymphocytes, the downregulation of immune-activating cytokines, and the upregulation of immunosuppressive molecules (Ogiya et al., 2016; Szekely et al., 2018; Manson et al., 2019; He et al., 2020). This might support the higher efficacy of checkpoint inhibitors in early-stage TNBC than in pretreated metastatic tumors (Adams et al., 2019a). This suggests a benefit to the use of immunotherapeutic options in earlier stages of the disease, associated with higher tumor immunogenic potential, rather than in the metastatic setting.

In fact, the expression of checkpoint molecules by TILs, supports the high expression of PD-1 and PD-L1 in triple-negative tumors (Wang and Liu, 2020) and correlations with good prognosis (increased pCR (Kitano et al., 2017)). The expression of PD-L1 in TILs could also be predictive of the response to checkpoint blockade immunotherapy against TNBC (Rizzo and Ricci, 2022). In fact, positive PD-L1 expression is currently used in clinics for the selection of patients who may benefit from anti-PD-1 (FDA, 2020a) and anti-PD-L1 (Roche, 2019a) treatments. Nonetheless, the results from the Impassion031 (Mittendorf et al., 2020) and KEYNOTE-522 (Schmid et al., 2020a) trials, demonstrating the efficacy of checkpoint inhibitors in combination with chemotherapy in early TNBC regardless of PD-L1 status, should prompt further research on the use of PD-L1 expression as a biomarker for patient stratification.

With the growing understanding of TILs as markers of breast tumors prognosis, in 2019, the WHO acknowledged the importance of stromal TILs as prognostic markers in breast cancers and changed their histological classification to include a category of TIL-rich Invasive Breast Carcinoma of No Special Type (Hoon Tan et al., 2020). Nevertheless, harmonization of TILs and PD-L1 expression determination and scoring is needed, as immunotherapy becomes an increasingly valuable alternative for the treatment of TNBC patients.

## Immunotherapy against triple-negative breast cancer

Immunotherapy entails a set of therapeutic approaches whose mechanism of action implies the stimulation of the immune system, in either an active (e.g., vaccines) or a passive (e.g., antibodies or immune modulators) way.

In breast cancer, immunotherapy has enabled great advances, particularly in the case of HER2-positive tumors. Patients with this subtype of breast cancer benefit from treatment with the anti-HER2 monoclonal antibody trastuzumab, both in the early and advanced stages, enjoying prolonged survival and lower toxicity (Lambertini et al., 2017). Trastuzumab’s success has encouraged the study of immunotherapy for other forms of breast cancer, such as TNBC. Higher levels of TNBC immunogenicity that are discussed in this review add to potential approaches such as immune checkpoint inhibitors, antibody-drug conjugates (ADCs), vaccines, and cellular therapies.

## Checkpoint inhibitors: From research to the clinics

Checkpoint inhibitors are monoclonal antibodies that target checkpoint proteins, inhibiting their immune-suppressive functions and prompting immune-mediated tumor cell death. In TNBC, the use of the checkpoint inhibitors pembrolizumab (anti-PD-1), atezolizumab (anti-PD-L1), avelumab (anti-PD-L1), and durvalumab (anti-PD-L1) have been studied in different regimens and for the treatment of different stages of the disease (Table 2). Blockage of CTLA-4 has also been considered for TNBC treatment, as its higher expression in patient tumors has been correlated with a better prognosis (Peng et al., 2020). However, this topic remains within the early stages of clinical research (phase I and II trials), and thus far, no approved anti-CTLA-4 molecules have been approved for use against TNBC. Therefore, this review focuses on the targeting of PD-1/PD-L1 interaction.

**TABLE 2:** Most relevant clinical trials for checkpoint inhibitors in TNBC, according to regimen and tumor stage.

	Trial	Phase	Objective	Intervention	Outcome/Results
**EARLY STAGE**	KEYNOTE-173 NCT02622074	Phase Ib	To evaluate the safety and efficacy of pembrolizumab in combination with six chemotherapy regimens as neoadjuvant treatment	Pembrolizumab + Nab-paclitaxel or paclitaxel + Carboplatin + Doxorubicin + Cyclophosphamide	Overall pCR = 60% (Schmid et al., 2020b)
	KEYNOTE-522 NCT03036488	Phase III	To evaluate the efficacy and safety of pembrolizumab plus chemotherapy vs. placebo plus chemotherapy as neoadjuvant therapy	Pembrolizumab or Placebo + Carboplatin + Paclitaxel + (Doxorubicin or Epirubicin) + Cyclophosphamide	pCR pembrolizumab + chemo = 64.8% pCR placebo + chemo = 51.2% a difference of 13.6% in pCR PD-L1 positive population did not have a significant increase in pCR (Schmid et al., 2020a)
	I-SPY2 NCT01042379	Phase II	To evaluate the efficacy of the combination of pembrolizumab and chemotherapy vs. chemotherapy alone	(Pembrolizumab +) Paclitaxel + Doxorubicin + Cyclophosphamide	pCR = 60% (vs. 22% with chemotherapy alone) (Nanda et al., 2020)
	GeparNuevo NCT02685059	Phase II	To evaluate response rates of neoadjuvant treatment of sequential chemotherapy and checkpoint inhibitor	Durvalumab or Placebo + Nab-paclitaxel + Epirubicin + Cyclophosphamide	pCR not significantly different between groups (Karn et al., 2020)
	Impassion031 NCT03197935	Phase III	To evaluate the efficacy and safety of neoadjuvant treatment of chemotherapy plus atezolizumab vs. chemotherapy plus placebo	Atezolizumab or Placebo + Doxorubicin + Cyclophosphamide + Nab-paclitaxel	pCR = 58% (vs. 41% with chemotherapy alone) In PD-L1 positive patients: pCR = 53% (vs. 37% with chemotherapy alone) (Mittendorf et al., 2020)
	NeoTRIPaPDL1 NCT02620280	Phase III	To compare the efficacy of chemotherapy plus atezolizumab vs. chemotherapy alone	(Atezolizumab +) Nab-paclitaxel + Carboplatin	pCR not significantly different between groups (ASCO, 2020)
**ADVANCED OR METASTATIC**	KEYNOTE-012 NCT01848834	Phase Ib	To evaluate the efficacy and safety of pembrolizumab in patients with advanced TNBC	Pembrolizumab	ORR = 18.5% (Nanda et al., 2016)
	KEYNOTE-086 NCT02447003	Phase II	To evaluate the efficacy and safety of pembrolizumab monotherapy as first-line or above treatment in patients with metastatic TNBC	Pembrolizumab	First-line: ORR = 21.4% (Adams et al., 2019a) Second-line or above: ORR = 5.3% (Adams et al., 2019b)
	PCD4989g NCT01375842	Phase I	Dose escalation study to evaluate the safety and clinical activity of atezolizumab monotherapy in patients with metastatic TNBC	Atezolizumab	As first-line: OS = 17.6 months; ORR = 24% As second-line or above: OS = 7.3 months; ORR = 6% (Emens et al., 2019)
	JAVELIN Solid Tumor NCT01772004	Phase Ib	Dose escalation study to evaluate safety and clinical activity of avelumab in patients with locally advanced or metastatic TNBC	Avelumab	ORR = 5.2% (Dirix et al., 2017)
	KEYNOTE-355 NCT02819518	Phase III	To compare the safety and efficacy of pembrolizumab plus chemotherapy vs. placebo plus chemotherapy in the treatment of patients with locally recurrent inoperable or metastatic TNBC who had been not previously treated with chemotherapy	Pembrolizumab or Placebo + Nab-paclitaxel or Paclitaxel or Gemcitabine or Carboplatin	PFS = 9.7 months (4.1 months longer than chemotherapy alone) (Cortes et al., 2020)
	IMpassion130 NCT02425891	Phase III	To evaluate safety and efficacy of the combination atezolizumab plus nab-paclitaxel vs. placebo plus nab-paclitaxel in patients with locally advanced or metastatic TNBC who had not received prior therapy for metastatic breast cancer	Atezolizumab or Placebo + Nab-Paclitaxel	OS = 21.3 months (vs. 17.6 months with chemotherapy alone) (Schmid et al., 2018)
	IMpassion131 NCT03125902	Phase III	To evaluate the efficacy and safety of atezolizumab plus paclitaxel vs. placebo plus paclitaxel in patients with previously untreated, locally advanced or metastatic TNBC	Atezolizumab or Placebo + Paclitaxel	No improvement in PFS or OS compared to paclitaxel alone (Miles et al., 2021)

Due to the relevance of anti-PD-1 and anti-PD-L1 antibodies in the treatment of TNBC, the density of PD-L1 expression as predictive of response has been clinically assessed across trials. Overall, a positive correlation was identified between PD-L1 expression and response outcomes, confirming the importance of PD-L1 assessment as a biomarker in TNBC (Emens et al., 2019; Schmid et al., 2020b; Karn et al., 2020). These results culminated in the approval of two checkpoint inhibitors accompanied by PD-L1 diagnostic tests, as previously mentioned (FDA, 2020a; Roche, 2020). Nonetheless, in the KEYNOTE-522 trial (Table 2), PD-L1 expression was not confirmed as a response predictor. Although PD-L1 status is usually measured through pre-treatment tumor biopsy, the PCD4989g trial demonstrated a significant increase in expression following treatment, suggesting that checkpoint therapy may promote tumor-specific T-cell activation and PD-L1 expression (Emens et al., 2019). This reinforces the limitations in trials designs, particularly the differences in PD-L1 assessment between studies, and the variety of drugs used in chemotherapy regimens, which may influence the tumor immune landscape (Emens et al., 2019). Other biomarkers were recently assessed in the clinical trial GeparNuevo, which was able to demonstrate that TMB and selected immune signatures (*CXCL9*, *CCL5*, *CD8A*, *CD80*, *CXCL13*, *ID O 1*, *PDCD1*, *CD274*, *CTLA4*, and *FOXP3* genes) have independent predictive value for chemotherapy, with and without durvalumab, in early TNBC (Karn et al., 2020).

Neoadjuvant chemotherapy induces tumor cell death, thus increasing antigen presentation, stimulating immunity and decreasing immunosuppression, which leads to changes in the immune cell composition of the tumor microenvironment (Denkert et al., 2018), suggesting that a possible benefit arising from combined regimens of immunotherapy following chemotherapy (Luen et al., 2019). It is then open to investigation whether immune blockage therapy is more beneficial as first-line therapy, or as a second, if it is beyond choice or combination strategies may be more effective than immune monotherapy, and to the identification of the disease setting that could most benefit the most from these approaches.

### First-line therapy vs. beyond

As neoadjuvant chemotherapy is standard for TNBC, most immune regimens are studied as second-line or later options. Nonetheless, the addition of a checkpoint inhibitor to chemotherapy showed significant clinical benefit in previously untreated patients with metastatic TNBC with PFS of 9.7 months for pembrolizumab (Cortes et al., 2020) and 7.5 months for atezolizumab therapies (Necela et al., 2015). The latter results were obtained from the Impassion130 study (Table 2) that prompted accelerated FDA and EMA approval of atezolizumab in combination with nab-paclitaxel for first-line treatment of PD-L1-positive patients with locally advanced and metastatic TNBC, the first immunotherapy approved for this subtype of breast cancer, in 2019 (Roche, 2019b). Recently, a second interim analysis for this study confirmed the clinical benefit of the combination of atezolizumab and nab-paclitaxel in PD-L1-positive tumors and its tolerable safety profile, despite the non-significant difference in OS observed in the intention-to-treat population (Schmid et al., 2020c). However, due to the modest results and the evolution of the landscape in the treatment of TNBC, in August 2021, the company decided to withdraw from accelerated approval of this combination for the treatment of adults with unresectable locally advanced or metastatic TNBC in PD-L1-positive tumors with FDA (Roche, 2020).

Modest results continued to be observed for checkpoint inhibitor monotherapy in first-line metastatic TNBC with the PCD4989g phase I study, which reported an ORR of 24% (Emens et al., 2019), and the KEYNOTE-173 trial, reporting an ORR of 21.4% and a PFS of only 2.1 months (Adams et al., 2019a). The combination of durvalumab with standard chemotherapy for first-line neoadjuvant treatment in early TNBC did not result in a significantly improved pCR relative to chemotherapy alone (Loibl et al., 2019). Overall, the clinical benefit of first-line immunotherapy in TNBC remains unclear, as more information about its effectiveness in previously untreated patients is needed.

### Monotherapy vs. combined regimens

The first study to evaluate the efficacy of checkpoint inhibition in TNBC was the KEYNOTE-012 trial (Table 2), which assessed the clinical activity of single-agent pembrolizumab in heavily pretreated patients with PD-L1-positive metastatic TNBC. An acceptable ORR of 18.5% (Nanda et al., 2016) was demonstrated, later confirmed in the cohort b KEYNOTE-086 trial, with an ORR of 21.4% and a median duration of response of 18 months (Adams et al., 2019a). Atezolizumab monotherapy showed similar results in the PCD4989g trial, with an ORR of 24% and a median OS of 17.6 months as first-line therapy (Emens et al., 2019). Single-agent durvalumab had a modest ORR of 5.2% in advanced or metastatic TNBC (Dirix et al., 2017).

However, the largest benefit of checkpoint inhibitor therapy arises from its combination with chemotherapy. This combination is hypothesized to increase tumor cell death by adding a second mechanism of action to standard cytotoxic agents and by taking advantage of the immune modulating effects of standard therapies (Denkert et al., 2018; Luen et al., 2019; Ji et al., 2021). Combination of atezolizumab with either paclitaxel (Miles et al., 2021) or carboplatin and nab-paclitaxel (ASCO, 2020) did not result in improvements in efficacy or safety outcomes relative to chemotherapy alone. Nonetheless, clinical benefit was achieved when the anti-PD-L1 antibody was combined with nab-paclitaxel. As mentioned before, this combination is approved for the first-line treatment of PD-L1-positive patients with locally advanced and metastatic cancer (Roche, 2019a; Roche, 2020). The extension of the approval to early and locally advanced stage was proposed after the Impassion-031 study resulted in a 53% pCR in patients in the atezolizumab group compared to 37% in the group treated with chemotherapy alone (Mittendorf et al., 2020). However, in June 2021, this application was withdrawn by the company, after EMA questioned the clinical benefits of the proposed regimen (EMA. Tecentriq, 2021).

As for anti-PD-1 antibody pembrolizumab, its simultaneous combination with the chemotherapeutic regimen resulted in increased benefit (Schmid et al., 2020a; Schmid et al., 2020b; Nanda et al., 2020), with pCR rates up to 38% higher than chemotherapy alone (Nanda et al., 2020). In fact, it has been approved by the FDA since November 2020 for the treatment of patients with locally recurrent unresectable or metastatic TNBC whose tumors express PD-L1 (CPS ≥10) (FDA, 2020a). and as neoadjuvant treatment, in addition to, since July 2021, use as a single agent in an adjuvant setting after surgery, in patients with early-stage high-risk TNBC (FDA, 2019). As for anti-PD-1 antibody pembrolizumab, its simultaneous combination with chemotherapeutic regimens resulted in increased benefit (Schmid et al., 2020a; Schmid et al., 2020b; Nanda et al., 2020), with pCR rates up to 38% higher than for chemotherapy alone (Nanda et al., 2020).

These results demonstrated the benefit of combination regimens and the need for the optimization of such therapeutic strategies that may see their efficacy increased from the use of checkpoint inhibitors together with specific chemotherapeutic agents.

### Early-Stage vs. advanced and metastatic

Early-stage TNBCs are more immunogenic than metastatic or heavily treated tumors (Szekely et al., 2018; Blackley and Loi, 2019; Hutchinson et al., 2020). Higher TMB levels and the increased presence of TILs translate into clear benefit of immunotherapy in early settings of the disease (Table 2). In fact, two major phase III trials, KEYNOTE-522 (Schmid et al., 2020a) and Impassion-031 (Mittendorf et al., 2020), demonstrated that the combination of a checkpoint inhibitor with chemotherapy improved pCR in 13.6 and 17% of patients, respectively, relative to standard chemotherapy. This was the basis for the approval of pembrolizumab in the treatment settings mentioned in the previous section (FDA, 2019). This benefit was corroborated by other studies (Nanda et al., 2017; Schmid et al., 2020b), although the GeparNuevo (Loibl et al., 2019) and NeoTRIPaPDL1 (ASCO, 2020) trials, in which a non-significant difference in pCR was found between groups, did not corroborate it. Nevertheless, patients in the window period cohort of the GeparNuevo trials, before starting chemotherapy, achieved significantly higher pCR after durvalumab treatment, relative to chemotherapy alone, than patients outside the window cohort (51 vs. 37.9%, respectively) (Loibl et al., 2019). This suggests that durvalumab may promote TIL migration to the tumor even before chemotherapy is administered

The study of checkpoint inhibitors in advanced and metastatic TNBC has occupied the majority of clinical trials so far (Rizzo et al., 2021). For previously treated patients, it is important to understand the impact of previous therapy in the immune composition of the tumors, as it may dictate the success or failure of adding an immunotherapeutic agent. Thus, the assessment of immune biomarkers plays a particularly important role in this setting. In this respect, the Impassion130 trial demonstrated that evaluation of PD-L1 positivity should be performed in tissue from the primary tumor instead of metastasis samples (Rugo et al., 2019). PD-L1 expression is mandatory for the selection of patients who may receive a combination of atezolizumab and nab-paclitaxel (Roche, 2019a) or pembrolizumab and chemotherapy (FDA, 2020a), which are currently approved by the regulatory authorities for patients with advanced or metastatic TNBC.

### Safety

Checkpoint inhibition leads to the activation of the immune system, which may not always be tumor specific, culminating in inflammatory toxicities in a variety of tissues, such as the skin, thyroid, liver, pancreas, colon, lung, heart, and central nervous system (Baraibar et al., 2019). In TNBC, the most common adverse events reported in clinical trials with checkpoint inhibitors are similar to those observed with chemotherapy and include anemia, neutropenia, nausea, alopecia, and liver function abnormalities, such as increased alanine aminotransferase (ALT) (Li et al., 2021). The incidence of treatment-related adverse events is clearly higher in combination regimens than in mono-immunotherapy (100% vs. 56–69%) (D’Abreo and Adams, 2019). Combined strategies are also associated with an increased frequency of grade 3/4 adverse events, mainly adrenal insufficiency, hepatitis, stomatitis, neutropenia, pyrexia, neuropathies, and pneumonitis (Li et al., 2021; Sternschuss et al., 2021).

Immune-related adverse events are usually controlled with the use of corticosteroids. Endocrinopathies are a common adverse event that deserves special consideration, as they may imply chronic hormone replacement therapy, thus impacting patients’ quality of life (Sternschuss et al., 2021). Infusion-related reactions are frequent both in immune and chemotherapy administration and are usually manageable low-grade events (Schmid et al., 2020b; Mittendorf et al., 2020). Overall, the safety profile of checkpoint blockage therapy is acceptable and manageable, and potential toxicities have to be balanced with the survival and durable response provided by the use of checkpoint inhibitors.

### Resistance

The emergence of mechanisms of resistance form another challenge in immune treatments, including in TNBC therapy (Luo et al., 2022). Both tumor-intrinsic and tumor-extrinsic mechanisms can lead to tumor resistance, particularly against checkpoint inhibitors (Kim et al., 2022).

The latter is related to the cellular composition of the tumor, where increased T-cell dysfunction (as a consequence of patient aging) (Sceneay et al., 2019) and the emergence of immunosuppressive cell populations after chemotherapeutic regimens (Zhang et al., 2021) play a major role in decreasing the efficacy of checkpoint therapy.

Tumor-intrinsic mechanisms are related to the activation of oncogenic pathways in the tumor microenvironment and they have been extensively studied. The clusters of genes implicated in inflammatory and immune responses are aberrantly expressed, allowing the tumor to escape the immune blockage of checkpoint inhibitors (Xiao et al., 2019). Combinatorial treatments with inhibitors of DNA methyltransferases (Wu et al., 2021) or activators of pathways involved in immune response (Loi et al., 2016) have been suggested to overcome this.

## Radiation: A potential complement to checkpoint inhibition

Radiation is also part of the arsenal available for TNBC management, especially after surgery, as adjuvant therapy addressing the minimal residual disease. Radiation has an immunosuppressive impact on cancer patients’ immune systems (Standish et al., 2008). Lymphopenia and decreased immune cell activity are possible consequences of radio-sensitivity of hematopoietic cells and lack of precision of traditional techniques. However, it may also stimulate immunity in the tumor microenvironment, which is now considered a stage in radiobiology principles. This effect depends on the radiation technique employed and dose, and the pre-existing composition of the tumor microenvironment (Demaria and Formenti, 2012). It is hypothesized that radiation increases tumors’ mutational load, prompting antigen presentation and T-cell activation, proliferation, and migration into the tumor microenvironment, and maybe even leading to a decrease in immune suppressors. Therefore, radiotherapy can function as an inductor of tumor immunity, complementing immunotherapy (Sharabi et al., 2015; Kang et al., 2016). Several clinical trials have been ongoing for a variety of cancers, with a number of phase I (NCT03366844), phase II (NCT02730130, NCT03464942, NCT03872505), and phase III (NCT02954874) studies testing for its combination with anti-PD-1 therapies against TNBC. These studies may be particularly important for patients with a higher risk of recurrence and lower sensitivity to chemotherapy.

A combination of immune and chemotherapy or radiotherapy has been proposed to enhance the expression of checkpoint molecules in tumors, making the tumor more sensitive to immunotherapy and increasing the efficacy of their inhibitors. In this regard, the phase II TONIC trial evaluated patients with metastatic TNBC who had received palliative chemotherapy and were subjected to induction therapies: irradiation of one metastatic lesion, or low-dose chemotherapy (doxorubicin, cyclophosphamide, or cisplatin). After induction treatment, patients received nivolumab, a monoclonal antibody targeting PD-1. Better responses were obtained in the doxorubicin (ORR 35%) and cisplatin (ORR 23%) groups, while the group without induction presented an ORR of 20%, in accordance with a demonstrated upregulation of PD-L1, T-cell activity, and genes related to an inflammatory response in the former group. Although the irradiation cohort showed an inferior ORR relative to the control no-induction group, the presence of TILs, the diversity of T-cell receptor repertoire, and inflammation-related gene signatures were higher than the ones observed for the no-induction cohort, indicating an immune modulation effect of radiation in the tumor microenvironment (Voorwerk et al., 2019).

Despite great advances arising from the use of checkpoint inhibitors, a considerable proportion of non-responders remain. Thus, alternative immunotherapy formats are under development.

## The quest for other forms of immunotherapy

### Vaccines

Vaccines are another strategy to promote antitumoral immune activity. Traditionally, cancer vaccines are based on tumor-associated antigens (TAA), but because TAAs are self-antigens, immune cells that recognize them are usually eliminated in the body’s maturation process. Further, the immunosuppressive mechanisms in the tumor microenvironment represent an additional barrier to this technology. Efforts to overcome these issues have been made, such as aiming at neoantigens instead of TAAs or combining vaccines with checkpoint inhibitors. Therefore, some therapeutic vaccines have demonstrated efficacy, long-term immune memory, and safety in a variety of tumors (Hollingsworth and Jansen, 2019). The strategies in development for TNBC are summarized in Table 3.

**TABLE 3:** Alternative immunotherapeutic approaches against TNBC.

Immune approach	Type of strategy	Rationale/Regimen	Clinical trial identifier
VACCINES	Peptide vaccine	Folate receptor alpha (overexpressed in TNBC cells (Necela et al., 2015)) peptide vaccine	NCT03012100
		AE37 (li-key HER2/neu hybrid) peptide vaccine + pembrolizumab	NCT04024800
		Neoantigen peptide vaccine + nab-paclitaxel + durvalumab + tremelimumab	NCT03606967
		PVX-410 multi-peptide vaccine (targeting TAAs XBP1, CD138 and CS1 (OncoPep, 2021)) + pembrolizumab	NCT03362060
		Galinpepimut-S peptide vaccine (targets the Wilms Tumor 1 protein) + pembrolizumab	NCT03761914
		P10s-PADRE (carbohydrate mimetic peptide P10s fused to the pan HLA DR-binding epitope - PADRE - peptide) + standard neoadjuvant chemotherapy	NCT02938442
	mRNA vaccine	Nanoparticle-containing mRNA, coding for the tumor antigen MUC1, overexpressed in TNBC (Siroy et al., 2013)	NCT00986609
		Liposome formulated vaccine based on the identification of individualized tumor-specific mutations by NGS and on-demand RNA manufacturing platform	NCT02316457
	DNA vaccine	Neoantigen DNA vaccine (designed based on advanced sequencing techniques and epitope prediction algorithms (Li et al., 2017b)) + durvalumab	NCT03199040
	Adenoviral cancer vaccine	Vaccine-Based Immunotherapy Regimen (VBIR-2): chimpanzee adenovirus expressing TAAs + tremelimumab + sasanlimab	NCT03674827
	Dendritic cell vaccine	Dendritic cell vaccine against Her2/Her3 + cytokine modulation (CKM) regimen + pembrolizumab	NCT04348747
		Dendritic cell vaccine loaded with cyclin B1, WT1, and CEF (overexpressed in TNBC (O’Shaughnessy et al., 2016)) + neoadjuvant chemotherapy	NCT02018458
		Tumor neoantigen autologous dendritic cell	NCT04105582
	Others	BN-Brachyury (transcription factor) vaccine + anti-PD-L1 and anti-TGF-β fusion protein	NCT04296942
		Adagloxad simolenin vaccine (tumor-associated carbohydrate antigen, covalently linked to the carrier protein KLH) (Huang et al., 2020)	NCT03562637
ADOPTIVE CELL THERAPY	Single therapy	TIL autologous therapy with lifileucel (a centrally manufactured TIL infusion product (Sarnaik et al., 2020))	NCT04111510
	Dendritic and cytokine-induced killer cells + chemotherapy	Dendritic and cytokine-induced killer cells + cyclophosphamide combined thiotepa (a preparative treatment prior to autologous cell transplantation (European Medicines Agency, 2020)) + carboplatin	NCT01395056
		Dendritic and cytokine-induced killer cells + cyclophosphamide	NCT01232062
	Natural killer and cytotoxic T lymphocytes + chemotherapy	ALECSAT therapy (Autologous Lymphoid Effector Cells Specific Against Tumor cells) (Cytovac, 2019) + carboplatin + gemcitabine	NCT04609215
	Autologous gene-edited T lymphocytes + checkpoint inhibitor	Autologous CD8 and CD4 T cells engineered to express a T cell receptor specific for a neoantigen from the patient’s tumor (Sennino et al., 2019) + nivolumab	NCT03970382
	T cells + chemotherapy	Autologous T cells targeting mesothelin (expressed in tumor cells of TNBC (Tozbikian et al., 2014)) + cyclophosphamide	NCT02792114
CAR-T cells	Allogeneic CAR-T cells	Allogeneic CAR-T cells targeting NKG2DL, a natural killer cells ligand expressed in tumor cells and involved in immunosuppressive mechanisms	NCT04107142
	Anti-MUC1 CAR-T + chemotherapy	Autologous CAR-T cells targeting MUS-1, a mucin glycoprotein overexpressed in TNBC, particularly in the basal-like tumors (Siroy et al., 2013) + cyclophosphamide + fludarabine	NCT04025216
ANTIBODY-DRUG CONJUGATES	Ladiratuzumab vedotin	Anti-LIV-1 humanized IgG1 antibody linked to monomethyl auristatin E, by a cleavable protease + pembrolizumab	NCT03310957
		Anti-LIV-1 humanized IgG1 antibody linked to monomethyl auristatin E, by a cleavable protease + several regimens of chemo and immunotherapy combinations	NCT03424005
	Glembatumumab vedotin	Glembatumumab antibody linked to monomethyl auristatin E via a protease sensitive linker + capecitabine	NCT01997333
	Sacituzumab govitecan	Anti-Trop-2 (anti-humanized antitrophoblast cell-surface antigen 2) antibody with SN-38, linked by a cleavable CLA2 linker	NCT04595565 NCT02574455
		Anti-Trop-2 (anti-humanized antitrophoblast cell-surface antigen 2) antibody with SN-38, linked by a cleavable CLA2 linker + pembrolizumab	NCT04230109 NCT04468061
		Anti-Trop-2 (anti-humanized antitrophoblast cell-surface antigen 2) antibody with SN-38, linked by a cleavable CLA2 linker + atezolizumab	NCT04434040

### Cell therapy

Cell therapy in the form of tumor-specific lymphocytes is also being explored, as cytotoxic cells express receptors on their surface that are specific to a given antigen, providing a potent and directed immune response. The main drawback of this approach is, therefore, the difficulty in choosing the correct target, especially in solid tumors, which are less immunogenic. However, some options have been adopted for TNBC, which include the adoptive transfer of both autologous or allogenic cells, and chimeric antigen receptor T-cells (CAR-Ts) (Guevara-hoyer et al., 2020).

Adoptive cell therapy consists of the transfer of activated immune cells to the patient. These cells can be harvested from the patient’s blood or tumor tissue, expanded *ex vivo,* and re-administered (autologous), or they can be taken from a different donor (allogeneic). The manipulation of immune cells outside the patients’ body may include expansion, activation, or engineering, undertaken with the purpose of prompting these cells to recognize specific tumor antigens and eliminate tumor cells. Despite the challenges associated with the manufacture of adoptive cell therapy, and the delivery and regulatory issues, the positive results found for cancer treatment, including solid tumors, are promising in the potential benefit for immunogenic cancers, such as TNBC (Morotti et al., 2021). In this regard, advances in the use of autologous cells in TNBC followed a 2017 study that analyzed TILs from a patient with a metastatic triple-negative tumor, where an immunogenic mutation capable of T-cell activation was identified and proposed for adaptive cell therapy (Assadipour et al., 2017). Since that time, adoptive cell therapy has been studied in the clinical in a variety of regimens, as summarized in Table 3.

CAR-T cells are T lymphocytes that have been engineered to express a molecule that is specific to a certain antigen, usually a small format antibody, such as single-chain variable fragments (scFv). As they are engineered to recognize such a specific target, CAR-T cells can be rapidly up-scaled to obtain a high number of antigen-specific T cells. In addition, their mechanism of action is independent of MHC (major histocompatibility complex) antigen presentation, which increases the number of target tumor cells. However, safety regarding these therapies is still a concern, with organ damage ensuing due to “on-target off-tumor toxicities” and intense (sometimes deadly) immune reactions (Sterner and Sterner, 2021). Still, clinical trials are ongoing for TNBC, either targeting TNBC antigens or other components of the tumor microenvironment, such as endothelial cells or fibroblasts (Dees et al., 2020; Xie et al., 2020). Currently ongoing or recently concluded clinical trials involving CAR-T cells in TNBC are summarized in Table 3.

### Antibody-drug conjugates

ADCs combine the specificity of monoclonal antibodies with the cytotoxic effect of potent small molecular weight drugs. For TNBC, three ADCs are under study (Nagayama et al., 2020) (Table 3). Ladiratuzumab vedotin (or SGN-LIV1A) is an anti-LIV-1 humanized IgG1 antibody linked to monomethyl auristatin E by a cleavable protease. LIV-1 is a transmembrane protein that is overexpressed in metastatic TNBC tumors, and monomethyl auristatin E is an antimitotic agent that is widely used in ADCs. When used as monotherapy in metastatic TNBC, the conjugated antibody demonstrated favorable efficacy (ORR of 32%) and a manageable safety profile, with the most common adverse events being nausea, fatigue, peripheral sensory neuropathy, and decreased appetite (Modi et al., 2018; Tsai et al., 2021). Because of its demonstrated immune stimulatory properties (Cao et al., 2018), ladiratuzumab vedotin has been studied in combination with pembrolizumab for patients with advanced or metastatic TNBC in two ongoing clinical trials (NCT03310957 and NCT03424005). Preliminary data showed an ORR of 54% in a cohort of 26 patients and a tolerable safety profile (HanHeather et al., 2020).

Glembatumumab vedotin (CDX-011) consists of a glembatumumab antibody that is linked to monomethyl auristatin E via a protease-sensitive linker. Glembatumumab targets glycoprotein NMB, and it is overexpressed in 40% of triple-negative tumors (Rose et al., 2010). The efficacy and safety of CDX-011 as monotherapy were evaluated in phase II clinical trials in patients with metastatic TNBC, overexpressing glycoprotein NMB (NCT01997333). However, the primary endpoint of the trial was not met (PFS was similar to the control group undergoing capecitabine treatment), and there was no reduced toxicity when compared to capecitabine alone (Vahdat et al., 2021).

**FIGURE 2:**
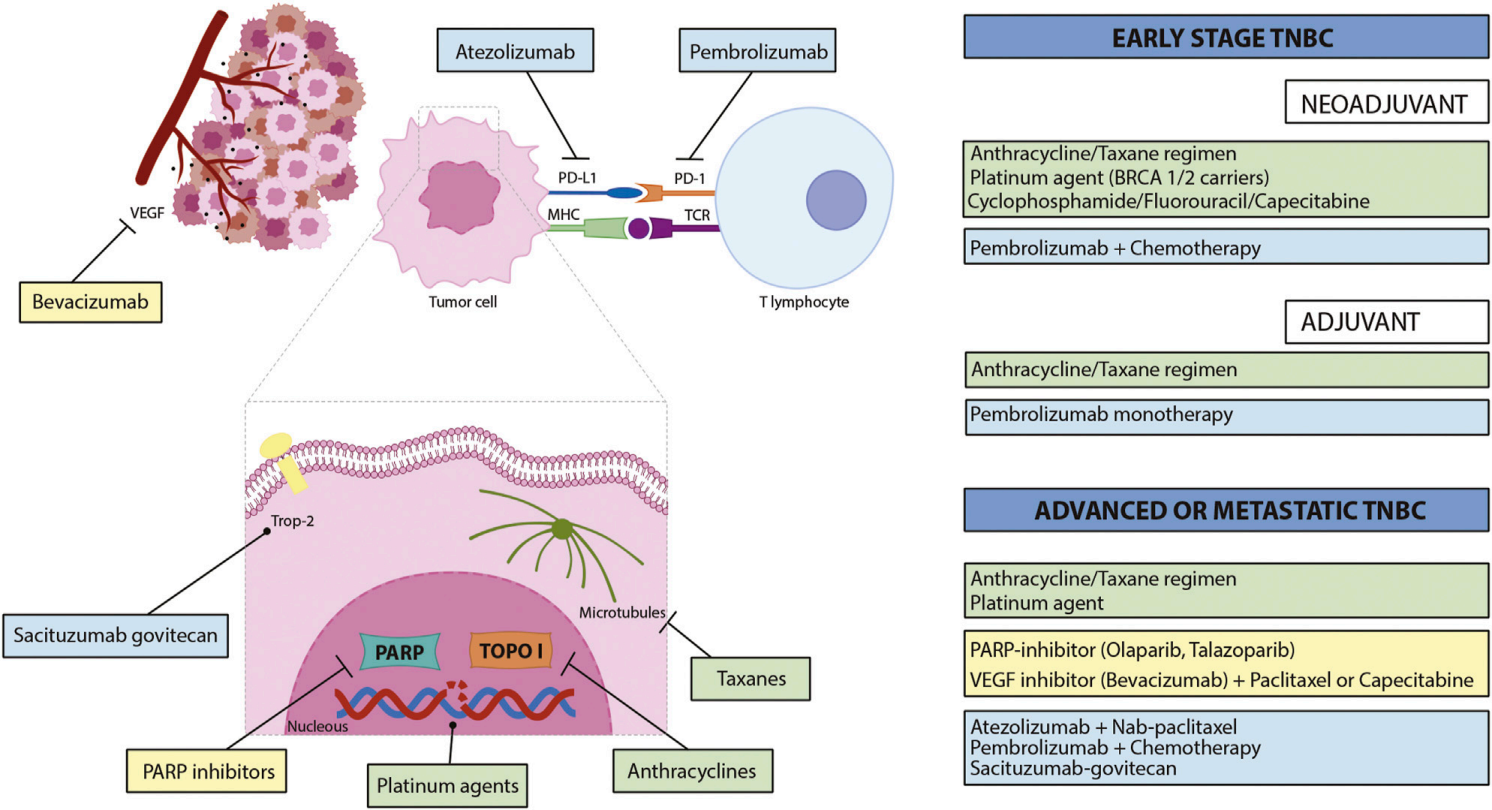
Currently approved pharmacological treatments of TNBC and their respective molecular targets. Green rectangles indicate chemotherapeutic regimens, yellow rectangles illustrate targeted therapies, and light blue rectangles refer to immunotherapies. PD-L1—programmed death-ligand 1; PD-1—programmed cell death protein 1; MHC—major histocompatibility complex: TCR—T-cell receptor; PARP—poly(ADP-ribose) polymerase; TOPO I—topoisomerase I.

Sacituzumab govitecan (IMMU-132) is an ADC that combines an anti-Trop-2 (anti-humanized antitrophoblast cell-surface antigen 2) antibody with SN-38, linked by a cleavable CLA2 linker. Trop-2 is a transmembrane molecule overexpressed in several epithelial cancers, such as TNBC (Huang et al., 2005), and SN-28 is an active metabolite of irinotecan and an inhibitor of topoisomerase I. Due to their demonstrated efficacy in a first phase I/II clinical trial, with an ORR of 33.3% and median response duration of 7.7 months (Bardia et al., 2019), in April 2020, FDA granted accelerated approval to sacituzumab govitecan for patients with metastatic TNBC who received at least two prior treatments for the metastatic setting (FDA, 2020b). Later, a confirmatory phase III trial compared the efficacy of ADC with single-agent chemotherapy in patients with relapsed or refractory metastatic TNBC. Patients treated with sacituzumab govitecan showed a significantly higher OS (12.1 vs. 6.7 months) and OR (35 vs. 5%) than patients treated with chemotherapy (Bardia et al., 2021). These results led to the approval by the FDA of sacituzumab govitecan for patients with unresectable locally advanced or metastatic TNBC, who have received two or more prior systemic therapies, at least one of them for metastatic disease (FDA, 2021). This last approval complements the currently available pharmacological treatments of TNBC, which are summarized in Figure 2.

At present, clinical trials with ADCs are testing their efficacy and safety as monotherapy and in combination with immunotherapy. These are summarized in Table 3.

## Conclusion and future prospects

Triple-negative breast cancer is an aggressive subtype of breast cancer. Advances in its treatment have been sporadic and have had unsatisfactory results, as chemotherapy remains the standard therapy in both neoadjuvant and adjuvant settings. The search for new targeted therapies has been intense, with PARP inhibitors leading the way. In this regard, both FDA and EMA approved olaparib and talazoparib for patients with germline BRCA mutations and HER2-negative locally advanced or metastatic breast cancer who have been previously treated with anthracycline and/or taxane. Other approaches have been more controversial, such as the use of angiogenesis inhibitors, as the clinical results have not been conclusive so far. Thus, TNBC remains an unmet medical need.

The immunogenic nature of triple-negative tumors represents a relevant opportunity for novel treatment. TNBCs have a higher density of TILs and TMB than other subtypes of breast cancer, encouraging the study of new biomarkers and forms of immunotherapy against the disease. The expression of PD-L1 in triple-negative tumors is already in clinical use as predictive of response to checkpoint inhibitors, but lack of harmonization between assays hampers its routine use. In the future, standardization of such methods, together with combined information from TMB, immune gene signatures, and TIL levels, will allow patients to benefit the most from the potential of immunotherapies against TNBC. The importance of immune biomarkers goes even further, as their role in diagnosis, patient stratification, and prognosis will fulfill the demands of the complexity of personalized medicine.

Regarding immunotherapy, checkpoint inhibitors demonstrated some clinical benefit to TNBC, particularly when used in combination with chemotherapy, by taking advantage of the effects of the latter in the immune landscape of tumors. Their efficacy was also demonstrably greater in the early stages of the disease than in the pretreated metastatic settings, due to the less immunogenic nature of metastasis. In this respect, two checkpoint inhibitors were approved: atezolizumab combined with nab-paclitaxel for first-line treatment of PD-L1 positive tumors with locally advanced and metastatic TNBC (EMA, 2019), pembrolizumab combined with chemotherapy for the treatment of patients with locally recurrent unresectable or metastatic TNBC whose tumors express PD-L1 (FDA, 2020), and pembrolizumab either combined with chemotherapy as neoadjuvant treatment or as a single agent as adjuvant therapy after surgery in patients with early-stage high-risk TNBC (FDA, 2021).

Other forms of immunotherapies are currently being studied, with highlight to ADCs, such as sacituzumab-govitecan, which has recently been approved for patients with unresectable locally advanced or metastatic TNBC who have received two or more prior systemic therapies, at least one of them for metastatic disease (FDA, 2021). Vaccines and cell therapy are under clinical research, with no demonstrated clinical benefit yet.

To improve the use of immunotherapy in the treatment of patients with TNBC, a better understanding of the interplay between the tumor with the immune system is needed, as well as the mechanism of action of the different immune therapies. The design of the clinical trials also requires improvement, as nowadays they tend to include distinct endpoints and different population sizes, which makes it difficult to analyze and compare the data obtained. The study of the combination of different immunotherapies or immune agents with other forms of therapy will need to evolve. Consciousness is growing that the stimulation of the tumor microenvironment causes a more favorable immunogenic state, improvement of the activity of immune therapeutic agents, and subsequent better clinical responses.
